# MytiLec-1 Shows Glycan-Dependent Toxicity against Brine Shrimp *Artemia* and Induces Apoptotic Death of Ehrlich Ascites Carcinoma Cells In Vivo

**DOI:** 10.3390/md17090502

**Published:** 2019-08-28

**Authors:** Imtiaj Hasan, A.K.M. Asaduzzaman, Rubaiya Rafique Swarna, Yuki Fujii, Yasuhiro Ozeki, Md. Belal Uddin, Syed Rashel Kabir

**Affiliations:** 1 Department of Biochemistry and Molecular Biology, Faculty of Science, University of Rajshahi, Rajshahi-6205, Bangladesh; 2 Department of Pharmacy, Faculty of Pharmaceutical Science, Nagasaki International University, 2825-7 Huis Ten Bosch, Sasebo, Nagasaki 859-3298, Japan; 3 Department of Life and Environmental System Science, School of Sciences, Yokohama City University, 22-2 Seto, Kanazawa-ku, Yokohama 236-0027, Japan

**Keywords:** apoptosis-related genes, Ehrlich ascites carcinoma, toxicity, lectin, MytiLec-1, *Mytilus galloprovincialis*

## Abstract

MytiLec-1, a 17 kDa lectin with β-trefoil folding that was isolated from the Mediterranean mussel (*Mytilus galloprovincialis*) bound to the disaccharide melibiose, Galα(1,6) Glc, and the trisaccharide globotriose, Galα(1,4) Galβ(1,4) Glc. Toxicity of the lectin was found to be low with an LC_50_ value of 384.53 μg/mL, determined using the *Artemia* nauplii lethality assay. A fluorescence assay was carried out to evaluate the glycan-dependent binding of MytiLec-1 to *Artemia* nauplii. The lectin strongly agglutinated Ehrlich ascites carcinoma (EAC) cells cultured in vivo in Swiss albino mice. When injected intraperitoneally to the mice at doses of 1.0 mg/kg/day and 2.0 mg/kg/day for five consecutive days, MytiLec-1 inhibited 27.62% and 48.57% of cancer cell growth, respectively. Antiproliferative activity of the lectin against U937 and HeLa cells was studied by 3-(4,5-dimethylthiazol-2-yl)-2,5-diphenyltetrazolium bromide (MTT) assay in vitro in RPMI-1640 medium. MytiLec-1 internalized into U937 cells and 50 μg/mL of the lectin inhibited their growth of to 62.70% whereas 53.59% cell growth inhibition was observed against EAC cells when incubated for 24 h. Cell morphological study and expression of apoptosis-related genes (p53, Bax, Bcl-X, and NF-κB) showed that the lectin possibly triggered apoptosis in these cells.

## 1. Introduction

Over time, well-organized defense mechanisms have been evolved in living systems for their survival. Lectins are a group of structurally diverse glycan-binding proteins that take part in the defense mechanism of invertebrates by specifically recognizing foreign particles and as effector molecules [1]. Lectins can bind to cell-surface glycoconjugates present in organisms that result in cell agglutination. They are also responsible for various intracellular and intercellular cell signaling and signal transduction. These proteins are present in diverse organisms, implicated in many essential cellular and molecular recognition processes and play a number of physiological roles including immunomodulatory [2], antitumor [3,4], antifungal [5,6], antibacterial [7,8], and antiviral [9] activities.

Over the last 30 years, lectins from marine organisms have received much attention from researchers. Though lectins from marine species are relatively new, researchers are trying to reveal their characteristics and biological applications in living organisms. They are present in more than 300 species and most of their structures, amino acid sequences, and carbohydrate specificities have been determined [10].

Lectins from marine organisms are classified into many families: C-type lectins, P-type lectins, F-type lectins, galectins, intelectins, rhamnose-binding lectins, and R-type lectins [1,11,12,13]. MytiLec (formerly MGL), which is an α-galactose-binding lectin, was first placed in the R-type lectin family, though it did not possess the additional toxic domain, a characteristic feature of R-type lectins [14]. Later, a new family known as ‘mytilectin’ was introduced [8]. At present, there are four members in the mytilectin family. Identification and elimination of microbial pathogens are the suggested physiological functions of the members of this family, whereas distinct antimicrobial activities are considered to be their hallmarks. As the first member of the mytilectin family, an α-d-galactose binding mussel lectin from *Crenomytilus grayanus* (CGL) interacted with gram positive and gram negative bacteria [15,16]. The third and fourth member of the mytilectin family, MCL (from *Mytilus californianus*) and MTL (from *Mytilus trossulus*) also showed growth suppressive activities against different bacteria and fungi [1,17].

The second member, MytiLec, a 17-kDa polypeptide, was isolated from the bivalve *Mytilus galloprovincialis* [14]. Based on the transcriptome analysis, three isoforms (MytiLec-1, -2, and -3) of this protein have been reported so far [8,14], whereas the aerolysin-like domain was present in MytiLec-2 and -3. Despite of not having that domain, MytiLec-1 could inhibit bacterial growth like its counterparts [8] and similar to CGL, interacted with Gb3-containing glycosphingolipid-enriched microdomains on Burkitt’s lymphoma (Raji) cell surface to trigger apoptosis [18,19,20]. In this study, glycan-based cell regulatory activities of MytiLec-1 was observed using two different living systems, i.e., aquatic crustaceans (brine shrimp *Artemia* nauplii) and various cancer cells. Toxicity of MytiLec-1 was checked against brine shrimp with evidence to its ability to bind with glycans expressed on those. Previous reports on the anticancer activity of MytiLec-1 were based on in vitro studies. In this work, for the first time, in vivo antiproliferative activity of MytiLec-1 was checked against Ehrlich’s ascites carcinoma cell lines using Swiss albino mice. An effort was also made to partially elucidate the apoptotic pathway of this anticancer activity. In addition, antitumor effect of the lectin against U937 and HeLa cell lines was investigated in vitro.

## 2. Results

### 2.1. Purification and Confirmation of the Molecular Mass of MytiLec-1

**Figure 1:**
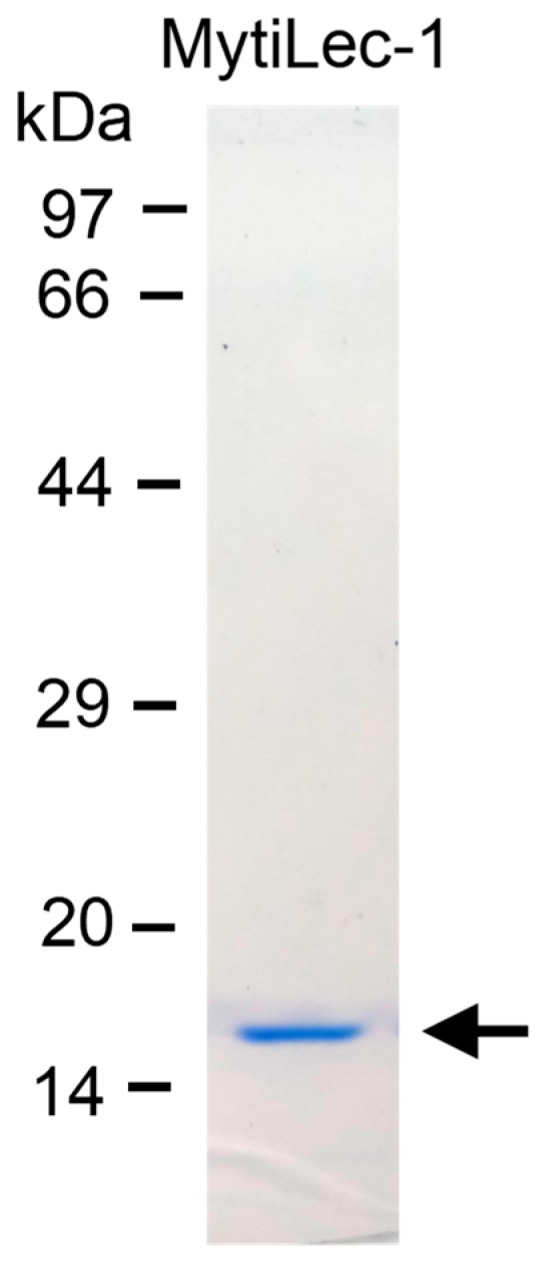
Purification of MytiLec-1 with a molecular weight of 17 kDa. Markers: Phosphorylase b (97 kDa), serum albumin (66 kDa), ovalbumin (44 kDa), carbonic anhydrase (29 kDa), trypsin inhibitor (20 kDa), and lysozyme (14 kDa).

Purified MytiLec-1 showed strong hemagglutination activity as it agglutinated human erythrocytes at the minimum concentration of 12 μg/mL. It migrated on SDS-PAGE as a single band with a molecular mass of 17 kDa (Figure 1).

### 2.2. Toxicity of MytiLec-1 against Brine Shrimp Artemia Nauplii

**Figure 2:**
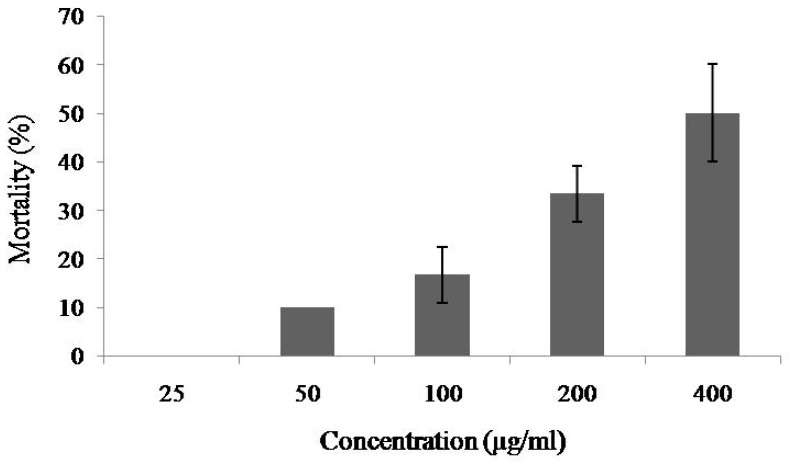
Percentage of mortality of brine shrimp nauplii treated with different concentrations of MytiLec-1. Data are expressed in mean ± S.D.

At the concentrations of 25–200 μg/mL of MytiLec-1, mortality rate of *Artemia* nauplii was 0% to 33%, and the rate increased to 50% when the concentration rose to 400 μg/mL and the LC_50_ value was determined to be 384.53 μg/mL (Figure 2).

### 2.3. Binding of FITC-Labeled Lectins to Artemia Nauplii Detected by Fluorescence Microscopy

**Figure 3:**
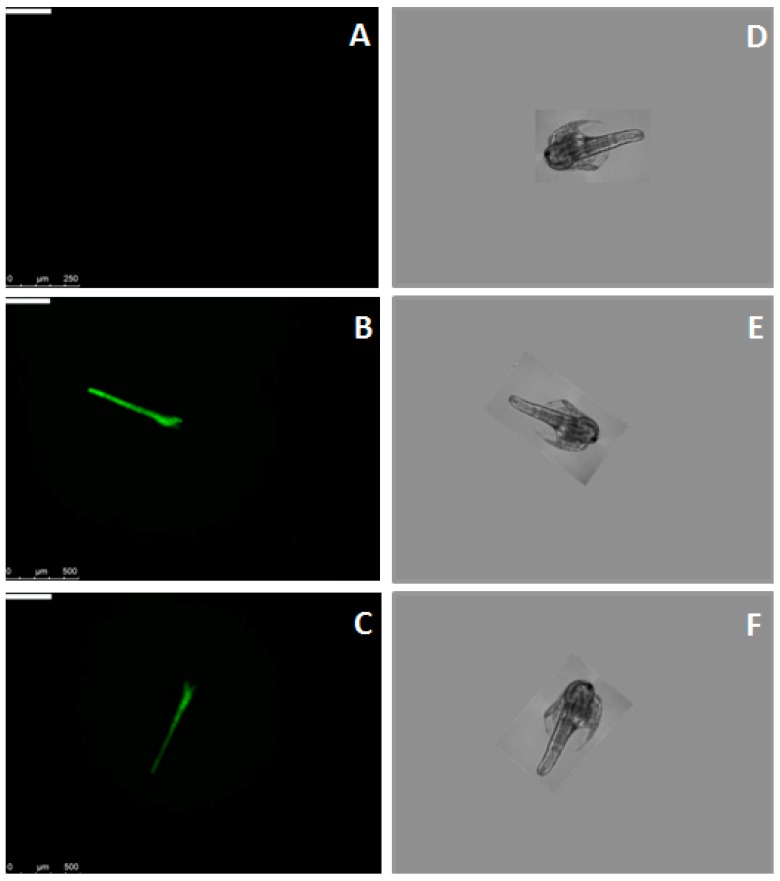
Binding of Fluorescein isothiocyanate (FITC)-labeled MytiLec-1 to *Artemia* nauplii detected by fluorescence and brightfield microscopy. Green color indicates the presence of FITC-labeled MytiLec-1 in the digestive tract of the animal. (**A**,**D**): FITC-BSA; (**B**,**E**): FITC-MytiLec-1; (**C**,**F**): FITC-MytiLec-1 with melibiose sugar.

Binding of MytiLec-1 to *Artemia* nauplii was confirmed by fluorescence microscopy. Figure 3A and 3B showed the absence and presence of green color of Fluorescein isothiocyanate (FITC)-BSA and FITC-MytiLec-1 in their digestive tracts, respectively. This binding was affected by the presence of melibiose (ligand sugar of MytiLec-1), as intensity of the green color became diminished (Figure 3C).

### 2.4. Agglutination of Ehrlich Ascites Carcinoma Cells

**Figure 4:**
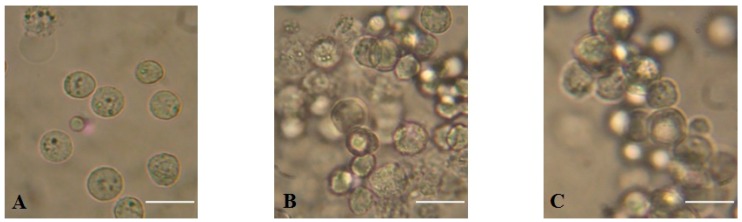
Agglutination of Ehrlich ascites carcinoma (EAC) cells by MytiLec-1. (**A**). Untreated control cells; (**B**). EAC cells treated with 50 μg/mLand (**C**). 100 μg/mL of MytiLec-1. Scale bar: 25 μm.

MytiLec-1 strongly agglutinated Ehrlich ascites carcinoma (EAC) cells at concentrations of 50 and 100 μg/mL (Figure 4), whereas the minimum agglutination concentration was 16 μg/mL.

### 2.5. In Vivo Antitumor Activity of MytiLec-1

**Figure 5:**
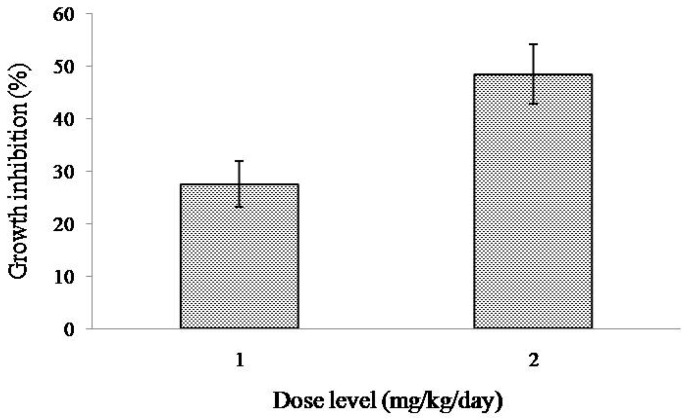
Inhibition of the growth of control and MytiLec-1 treated EAC cells. Data are expressed in mean ± S.D (*n* = 6).

When treated with intraperitoneal injection of MytiLec-1 for five days, growth of EAC cells in tumor-bearing Swiss albino mice became reduced comparing to EAC cells in untreated (or control) mice. At the doses of 1 and 2 mg/kg/day of MytiLec-1, around 28% and 49% cell growth inhibition were found (Figure 5).

### 2.6. Morphological Examination of Ehrlich Ascites CarcinomaCells

**Figure 6:**
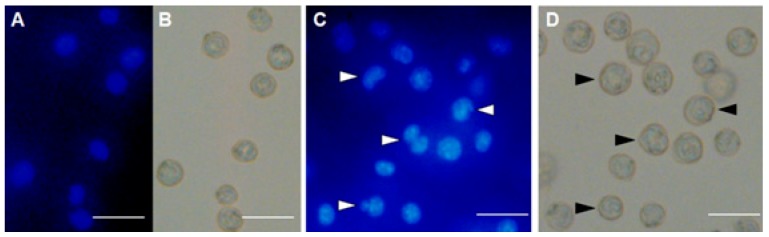
MytiLec-1 induces apoptotic morphological features in EAC cells. Cells were stained with Hoechst-33342 dye to be observed by fluorescence (**A**,**C**) and bright field microscopes (**B**,**D**). (**A**,**B**) Untreated control cells. (**C**,**D**) Cells treated with 50 mg/mL of MytiLec-1 for 24 h. Scale bar: 50 μm. White and black arrows represent cells undergoing apoptosis.

EAC nuclei from cells in the control group were found to be in round and normal shape (Figure 6A). Contrarily, MytiLec-1 treated cells showed characteristic morphological alterations (irregular shapes, nuclear condensation, and presence of apoptotic bodies) when observed by fluorescence (Figure 6B) and bright field microscopes (Figure 6C).

### 2.7. Expression of Apoptosis-Related Genes

**Figure 7:**
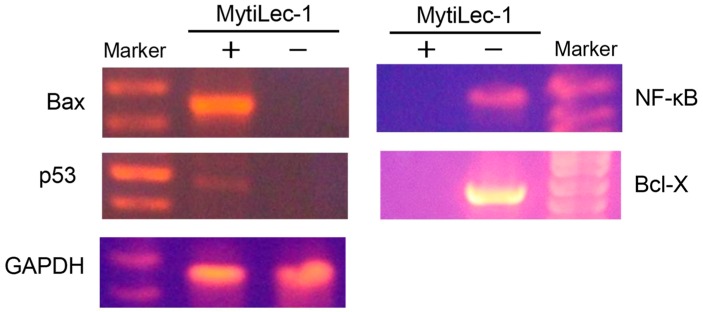
Amplification of apoptosis related genes. Total RNA was extracted from MytiLec-1 treated (+) and untreated (−) EAC cells and reverse transcription was performed. PCR reaction was carried out using primers specific for Bax, NF-κB, p53, Bcl-X, and GAPDH whereas products were separated on 1.5% agarose gel and stained with ethidium bromide.

The expression level of Bax was high in MytiLec-1 treated EAC cells although no expression was found in control EAC cells. Opposite results were found in case of Bcl-X and NF-κB genes. Expression of these genes became upregulated in control EAC cells and lectin-treated EAC cells showed no expression at all. Though it was very low, expression of p53 gene was observed in the lectin-treated cells. Expression of glyceraldehyde 3-phosphate dehydrogenase (GAPDH) was satisfactory to confirm the quality of mRNA from lectin-treated and control EAC cells (Figure 7).

### 2.8. Internalization of MytiLec-1 into U937 Cells

**Figure 8:**
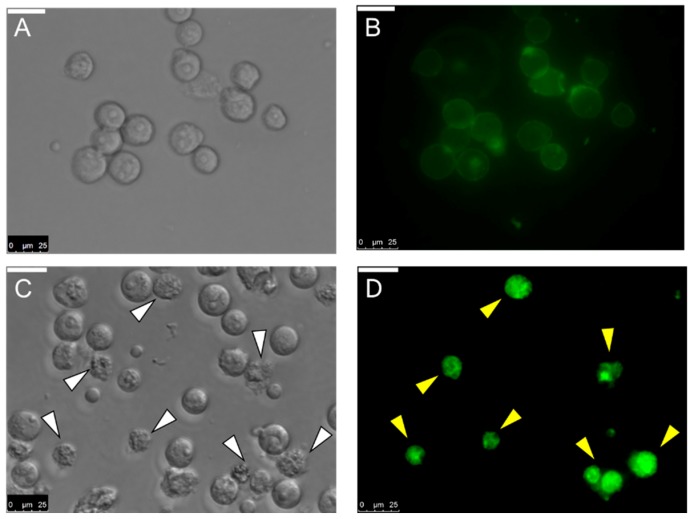
Internalization of MytiLec-1 into U937 cells. Cells were observed by phase contrast (**A**,**C**) and fluorescence microscopy (**B**,**D**). Incubation time: 5 min (**A**,**B**)and 1 h (**C**,**D**). Scale bar: 25 μm.

Not only binding with U937 cells, MytiLec-1 was observed to be internalized into those cells by fluorescence microscopy. Similar result had been found for Burkitt’s lymphoma cells where the lectin induced morphological changes in cells [19]. In this case, changes in cell morphology were observed after 1 h of incubation with MytiLec-1 (Figure 8).

### 2.9. In Vitro Antiproliferative Activity of MytiLec-1 against U937 and HeLa Cell Lines

**Figure 9:**
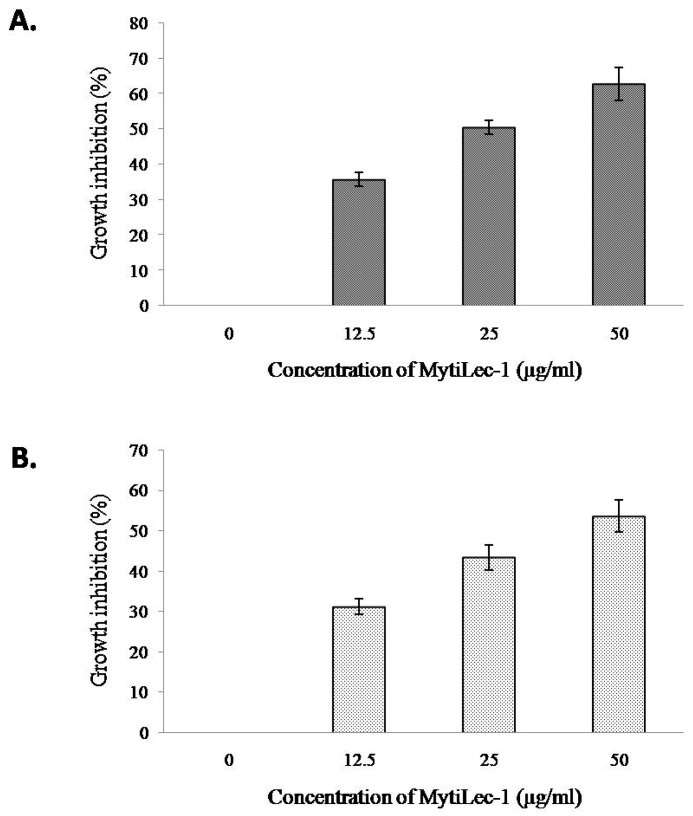
Growth inhibition of human cancer cells after treatment with MytiLec-1. Cell proliferation of U937 (**A**) and HeLa (**B**) cells was measured by an MTT assay (*n* = 3, mean ± S.D) after treating those with different doses of MytiLec-1.

MTT assay showed a dose dependent effect of MytiLec-1 against U937 and HeLa cells. U937 cells were slightly more susceptible to MytiLec-1 comparing to HeLa cells. Around 36–63% of cell growth inhibition of U937 cells was found at the protein concentration of 12.5–50 μg/mL (Figure 9A), whereas against HeLa cells, 32–54% of cell growth inhibition was observed (Figure 9B).

## 3. Discussion

MytiLec-1 showed mild lethality with an LC_50_ value of 384.53 μg/mL (Figure 2). In a previous study, CvL-2, another galactose-binding trimeric lectin from *Cliona varians*, was reported to have lower lethality (LC_50_ value of 850.1 μg/mL) since the Sea cucumber (*Holothuria grisea*) lectin HGL was highly toxic (LC_50_ value of 9.5 μg/mL) against *Artemia* nauplii [21]. Two other lectins, H-1 and H-2, from a marine sponge *Haliclona caerulea*, showed higher toxicity (LC_50_ value of 6.4 and 142.1 μg/mL, respectively) than MytiLec-1, putting it in a category of mildly toxic lectins [22].

It became evident that the lectin bound to the digestive tract of *Artemia* nauplii (Figure 3B). Heavily glycosylated digestive tract of nauplii might contain α-galactoside sugars that became specifically recognized by MytiLec-1 as addition of 0.1M melibiose [Gal(α1-6)Glc] sugar reduced the binding of FITC-labeled MytiLec-1 (Figure 3C). This finding was in line with the properties of previously reported lectins from the genus *Canavalia* [23]. Though plant lectins are usually more toxic than animal lectins, this result suggested that animal lectins follow similar mechanisms to interrupt the functions of gut cells of *Artemia* or to destroy those cells [24,25].

A number of marine lectins, including MytiLec-1, have been reported to bind the outer sugar chains of glycoconjugates on cancer cell surface and induce anti-cancer effects [4]. Members from the Mytilidae lectin family are fairly distributed in nature and many of the structural and biological properties of MytiLec have already been elucidated. It is a lectin with β-trefoil fold and specific binding property to Gb3 (Galα1-4Galβ1-4Glc). Antitumor activities and mechanism of action of both forms, natural and artificial, of MytiLec-1 has been studied in vitro against Burkitt’s lymphoma Raji cells [17,19,20,26]. As both animal studies and clinical studies are essential to check the possibility to utilize the lectin for chemotherapeutic treatment, this work attempted an in vivo study on Ehrlich’s ascites carcinoma. 

Ehrlich ascites carcinoma cells are the adapted ascites form of mammary adenocarcinoma cells. Many lectins agglutinated these cells in various concentrations and inhibited their growth [27,28,29,30]. It has long been established that cell membranes of EAC cells contain a family of α-d-galactosyl containing glycoproteins and glycolipids [31,32], which are ligand sugars of MytiLec-1. The lectin strongly agglutinated EAC cells (Figure 4) with a minimum concentration of 16 μg/mL. It had been reported that Jacalin, another α-d-galactosyl specific lectin from Jackfruit, agglutinated the same cells at a minimum concentration of 8 μg/mL [33].

After confirming the glycan-specific agglutination activity, the lectin was injected into Swiss albino mice to study its antiproliferative activity. At doses of 1 mg/kg/day and 2 mg/kg/day, MytiLec-1 showed around 28% and 49% of cell growth inhibition (Figure 5). A lectin from *Pisum sativum* exhibited growth inhibition rates of 63% and 44% against EAC cells at concentrations of 2.8 mg/kg/day and 1.4 mg/kg/day, respectively [34]. In the case of KRL-2 from *Kaempferia rotunda*, 41% and 59% of EAC cell growth inhibition was found at the doses of 3 mg/kg/day and 6 mg/kg/day [27]. At a fixed concentration of 100 μg/mL, plant extracts with lectin activity from *Ricinus communis* and *Amaranthus hybridus* led to 54 and 45% growth inhibition of EAC cells, respectively [35,36]. Considering these results, antiproliferative activity of MytiLec-1 to EAC cells is quite significant despite of its low toxicity to brine shrimp. In Figure 6, fluorescence and optical microscopy of EAC cells from the MytiLec-1 treated mice showed early-apoptotic morphological changes in shape and size along with membrane blebbing, cell shrinkage, nuclear condensation and formation of apoptotic bodies, comparing to round sized cells with regular nuclei from the control mice.

Apoptosis, a common type of programmed cell death, takes place in response to various cellular damages, stress, and molecular stimuli. This highly regulated process plays significant roles in the development and homeostasis of eukaryotic organisms. Induction of apoptosis by a lectin from *Kaempferia rotunda* has been reported to occur in EAC cells. Expression of Bax genes increased significantly with a marked decrease of Bcl-2 and Bcl-X genes [37]. Involvement of p53 genes had also become evident through its increased expression when two other plant lectins from *Geodorum densiflorum* and *Solanum tuberosum* inhibited the growth of EAC cells [28,30]. In p53-dependent mitochondrial pathways, p53 upregulates the transcription of pro-apoptotic genes, including Bax and Bak, and downregulates anti-apoptotic genes, including Bcl-2, Bcl-X, and Bcl-w, to augment apoptosis in many cancer cells [38]. In this study, a faint band of p53 was observed in MytiLec-1 treated cells, whereas the band for Bax was very prominent. Upon binding to Bcl-2 family proteins, p53 aids Bax to become available to transmit an apoptotic signal to mitochondria. Cytochrome c got released through the Bax-Bak oligomeric pores and switched on the caspase cascade, leading to apoptosis [39]. Figure 7 shows complete downregulation of the expression of anti-apoptotic gene Bcl-X and transcriptional factor NF-κB indicating a possible augmentation of the above process [40]. We also noticed the overexpression of Bax, which probably regulated the function of Bcl-X [28]. We should not rule out the possibility of a p53-independent pathway as Bax and Bak had been previously reported to trigger apoptosis in the absence of p53 [41,42,43] and both these pathways can run concurrently [39].

It had been reported that U937 and HeLa cells possess glycosphingolipids Gb3 as a cell surface receptor to bind with various molecules like lectins and toxins [44,45]. MytiLec-1 was previously reported to internalize into Burkitt’s lymphoma cells to cause cytotoxicity [19]. In the present study, the lectin showed the same tendency to incorporate into U937 cells (Figure 8). Another marine lectin, HOL-18 from Japanese black sponge (*Halichondria okadai*) also went inside of a number of cancer cells including HeLa, MCF-7, and T47D to inhibit their growth but did not response to Caco-2 cells [46]. A lactose-binding fungal lectin showed the same behavior but did not cause cytotoxicity of HeLa cells [47]. Therefore, MytiLec-1 perhaps specifically interacted with Gb3 to reduce the growth of both U937 and HeLa cells. The MTT assay also showed that U937 cells were more susceptible (63%) to MytiLec-1 than HeLa cells (54%) at the concentration of 50 μg/mL (Figure 9). Being a lot more toxic in nature, ricin (first member of the lectin with β-trefoil folding) triggered cell death in U937 cells in a much lower concentration [48,49]. Many other lectins from animal and plant sources had been reported to exert dose-dependent growth inhibitory effects against U937 and HeLa cell lines, proceeding to apoptosis and cell death [50,51,52,53].

## 4. Experimental Design

### 4.1. Materials

Melibosyl-agarose column was prepared by packing agarose gel immobilized melibiose (Galα1-6Glc) (EY Laboratories Inc., San Mateo, CA, USA) in Poly-Prep chromatography column (Bio-Rad Laboratories, Irvine, CA, USA). RPMI-1640 medium, fetal calf serum, and Hoechst-33342 were purchased from Sigma Aldrich (St. Louis, MO, USA). Penicillin-streptomycin was bought from Roche Diagnostics. Standard protein markers for SDS-PAGE were purchased from Takara Bio Inc. (Kyoto, Japan). Poly-L-lysine-coated slides used in this study were from MilliporeSigma (Darmstadt, Germany). All other chemical/reagents, each of the highest purity grades were from Wako Pure Chemical Co. (Osaka, Japan) and Sigma Aldrich (St. Louis, MO, USA).

### 4.2. Purification of Protein

*Mytilus galloprovincialis* mussels were commercially purchased from the local market of Yokohama, Japan. Gills and mantles are homogenized with 2-amino-2-(hydroxymethyl)propane-1,3-diol;hydrochloride (Tris-HCl) buffer (pH 7.4). The crude supernatant was applied on a melibiosyl-agarose affinity column and MytiLec-1 was eluted by using Tris-HCl buffer containing 100 mM of melibiose sugar [14]. Eluted MytiLec-1 was dialyzed against Tris-HCl buffer to remove the sugar. Purity of the protein was checked by using SDS-PAGE (sodium dodecyl sulfate polyacrylamide gel electrophoresis) in 16% (w/v) polyacrylamide gel as described by Laemmli [54].

### 4.3. Brine Shrimp Nauplii Lethality Assay

Toxicity of MytiLec-1 was checked using brine shrimp nauplii (*Artemia salina*) lethality assay [55]. Shrimp nauplii were placed in six vials (ten in each vial) and MytiLec-1 was added to the vials at final concentrations of 0.0, 25.0, 50.0, 75.0, 100.0, and 200.0 μg/mL. The volume of each vial was adjusted by adding artificial sea water. Artificial sea water was prepared by dissolving 38 g of NaCl in 1 L of distilled water. To adjust the pH to 7.0, sodium tetraborate salt was added to it. The vials were kept at 30 °C for 24 h under a light source. Three replicates were used for each experiment. Percentage of mortality of the nauplii was calculated for each concentration and LC_50_ value of MytiLec-1 was determined using Probit analysis [56].

### 4.4. Preparation of Fluorescein Isothioycanate (FITC)-Labeled Proteins

FITC-labeled MytiLec-1 was prepared by conjugating purified MytiLec-1 (2 mg) with NH_2_-reactive fluorescein isothioycanate (Dojindo Laboratories, Kumamoto, Japan) following the manufacturer’s protocol. A washing buffer (PBS, i.e., phosphate-buffered saline: 0.01M sodium phosphate buffer, 0.027M KCl and 0.15M NaCl, pH 7.4) was prepared. FITC-lectin was passed through a Sephadex G-25 column to remove unconjugated FITC molecules. The FITC-labeled MytiLec-1 was then dialyzed for 12 h against distilled water. Similarly, Bovine serum albumin (BSA) protein was labeled to FITC, which produced FITC-BSA.

### 4.5. Fluorescence Microscopy of Artemia Nauplii

*Artemia* nauplii were incubated with FITC-MytiLec-1, FITC-BSA (50 μg/mL) and FITC-MytiLec-1 (with 0.1M melibiose sugar) and kept overnight. The shrimp were then washed thrice in PBS, placed on slides, and examined using an optical and fluorescence microscopy (Olympus IX71, Seoul, Korea). 498 nm lasers were used for the excitation of FITC.

### 4.6. Experimental Animals and Ethical Clearance

Swiss albino mice were collected from the ICDDR’B (International Center for Diarrheal Diseases Research, Dhaka, Bangladesh). This investigation was officially recognized by the Institutional Animal, Medical Ethics, Biosafety, and Biosecurity Committee (IAMEBBC) for Experimentations on Animals, Human, Microbes and Living Natural Sources (Memo No. 55/320/IAMEBBC/IBSc), Institute of Biological Sciences (IBSc), University of Rajshahi, Rajshahi, Bangladesh.

### 4.7. Ehrlich Ascites Carcinoma Cell Agglutination

In this next step, 50 μL of hemagglutination buffer was prepared and used to check the agglutination of EAC cells. The protein solution (50 μL) was serially diluted in a titer plate and then 50 μL of 2% EAC cells in saline were added. The plate was shaken for 7 min by a microshaker as well as incubated at 34 °C for 60 min.

### 4.8. Determination of Growth Inhibition of Ehrlich Ascites Carcinoma Cells

EAC cells were maintained through an intraperitoneal transformation into the Swiss albino mice in every two weeks. Development of ascites carcinoma in all mice was confirmed by the increase of their weight (data not shown). Cells were collected from a mouse bearing 1-week old ascites tumors and their concentration was adjusted to 4× 10^6^ cells/mL with 0.9% normal saline. After counting cells using a haemocytometer, their viability was verified by 0.4% trypan blue exclusion assay. Further, 0.1 mL of tumor cells (99% viable) were injected intraperitonealy into each Swiss albino mouse. The mice were divided into three groups with a minimum number of six mice in each group. One group was kept as the control group and after 24 h, the other two groups of mice were treated with an intraperitoneal injection of MytiLec-1 at the doses of 1.0 and 2.0 mg/kg/day. After five days treatment, mice in each group were slaughtered on the sixth day. EAC cells from each mouse was harvested in 0.9% saline by intraperitoneal injection and counted by a haemocytometer. The total number of viable cells in every mouse of the treated groups was compared with those of the control group. The percentage of inhibition was calculated by using the following formula:

Percentage of inhibition = 100 − {(cells from MytiLec-1 treated mice/cells from control mice) × 100}

### 4.9. Examination of Morphological Alteration and Nuclear Damages of Ehrlich Ascites Carcinoma Cells by Hoechst Staining

Signs of apoptosis of EAC cells were morphologically observed without and with MytiLec-1 using an optical and fluorescence microscopy (Olympus IX71, Seoul, Korea). EAC cells were collected from the mice treated with and without MytiLec-1 (2.0 mg/kg/day) for five consecutive days and washed thrice with PBS. Cells were then stained with 0.1 μg/mL of Hoechst-33342 at 37 °C for 20 min in the dark and washed again with PBS to remove the unbound dye.

### 4.10. Isolation of RNA from Ehrlich Ascites Carcinoma Cells and Expression of Apoptosis-Related Genes

Tiangen Biotech reagent kit (Beijing, China) was used to isolate the total RNA from EAC cells collected from lectin-treated and lectin-untreated Swiss albino mice. Concentration and purity of the isolated RNAs were determined by a spectrophotometer at 260 and 280 nm. RNA quality was checked by 1.4% agarose gel electrophoresis as the gel was stained with 10 μg/mL of ethidium bromide and the bands were visualized with a gel documentation system (Cleaver Scientific Ltd., Rugby, UK). cDNA samples were prepared from the isolated RNA following the manufacturer’s protocol (Applied Biosystems, Walthum, MA, USA). Primer sequences used in this study are shown in Table 1.

The program for amplification reactions was fixed at 95 °C for 3 min, 94 °C for 30 s, 55 °C 30 s, 72 °C for 50 s, and 72 °C for 10 min. Eventually, it was held at 20 °C in the thermal cycler (GeneAtlas, Tokyo, Japan). Expression of a housekeeping gene (GAPDH) was checked to confirm the quality of mRNA of the lectin-treated and untreated samples. All PCR reactions were analyzed by 1.4% agarose gel electrophoresis in the presence of a 100 bp DNA ladder (Sigma) as marker.

### 4.11. Cell Culture

HeLa (cervical cancer cells) and U937 (myeloid leukemia cells) were obtained from the Riken Cell Bank, Tsukuba, Japan and were cultured in RPMI-1640 medium with 10% fetal calf serum, and 1% (v/v) penicillin–streptomycin, in 25 cm^2^ tissue culture flasks at a humidified atmosphere of 5% CO_2_ at 37 °C. Cells were sub-cultured at regular intervals whenever the confluence reached to 70–80%.

### 4.12. Incubation of Fluorescein Isothiocyanate (FITC)-Conjugated MytiLec-1 with U937 Cells

Next, 2 mg of MytiLec-1 was conjugated with NH_2_-reactive fluorescein Isothiocyanate (Dojindo Laboratories, Kumamoto, Japan) according to the manufacturer’s instructions. U937 cells were cultured, taken on 18 mm round cover slips, and incubated with 100 μg/mL of FITC-MytiLec-1 from 5 min to 1 h. Cells were fixed with 4% paraformaldehyde for 15 min and observed using a Leica TCS SP5 confocal microscope (Wetzlar, Germany).

### 4.13. 3-(4,5-Dimethylthiazol-2-yl)-2,5-Diphenyl Tetrazolium Bromide (MTT) Colorimetric Assay of Different Cancer Cell Lines

MTT colorimetric assay was performed to determine the proliferation of U937 and HeLa cells according to a previous report [27]. Cells (2 × 10^4^ in 150 μL RPMI 1640 media) were seeded in a 96-well flat bottom culture plate and incubated at 37 °C in a CO_2_ incubator for 24 h. Cells were then incubated again for 48 h in the absence and presence of various concentrations (50–12.5 μg/mL) of MytiLec-1. Media containing only U937 and HeLa cells were used as positive controls. After carefully draining the aliquot, 10 mM of PBS (180 μL) and MTT (20 μL, 5 mg/mL MTT in PBS) were added and incubated for 8 h at 37 °C. The aliquot was removed again and 200 μL of acidic isopropanol was added to every well and incubated again at 37 °C for 30 min. The absorbance was recorded at 570 nm by a culture plate reader. Three wells were employed for each concentration and the following equation was followed to calculate the cell proliferation inhibition ratio:

Proliferation inhibition ratio (%) = {(A − B) × 100}/A

where A is the OD_570_ nm of the cellular homogenate (control) without MytiLec-1 and B is the OD_570_ nm of the cellular homogenate with MytiLec-1.

### 4.14. Statistical Analysis

For each of the studied parameters, experimental results were presented as mean ± standard error (SE) for three replicates. Data were subjected to one-way analysis of variance (ANOVA) followed by Dunnett’s test, using the SPSS Statistics software package, v. 10 (IBM Corporation, Armonk, NY, USA). Differences with *p* < 0.05 were considered as statistically significant.

## 5. Conclusions

In this study, it became evident that MytiLec-1 was mildly cytotoxic, strictly glycan-dependent in its actions, and effectively inhibited the growth of different cancer cells in vivo and in vitro. The protein activated Bax/Bak genes eventually caused apoptosis, possibly through a p53-dependent pathway. MytiLec-1 can be considered significantly active against cancer cells, but further studies are necessary to resolve the explicit intrinsic molecular mechanism of action of MytiLec-1.

## Figures and Tables

**Table 1:** List of primers.

Primer	Forward	Reverse
GAPDH	GTGGAAGGACTCATGACCACAG	CTGGTGCTCAGTGTAGCCCAG
Bax	CCTGCTTCTTTCTTCATCGG	AGGTGCCTGGACTCTTGGGT
Bcl-X	TTGGACAATGGACTGGTTGA	GTAGAGTGGATGGTCAGTG
NF-κB	AACAAAATGCCCCACGGTTA	GGGACGATGCAATGGACTGT
p53	GCGTCTTAGAGACAGTTGCCT	GGATAGGTCGGCGGTTCATGC
